# A novel gene expression test method of minimizing breast cancer risk in reduced cost and time by improving SVM-RFE gene selection method combined with LASSO

**DOI:** 10.1515/jib-2019-0110

**Published:** 2020-12-29

**Authors:** Madhuri Gupta, Bharat Gupta

**Affiliations:** Department of Computer Engineering and Information Technology, ABES Engineering College, Ghaziabad, Uttar Pradesh, India; Department of CS&IT, Jaypee Institute of Information Technology, Noida, Uttar Pradesh, India

**Keywords:** gene expression analysis, gene selection, Least Absolute Shrinkage Selector Operator (LASSO), machine learning, regression

## Abstract

Breast cancer is the leading diseases of death in women. It induces by a genetic mutation in breast cancer cells. Genetic testing has become popular to detect the mutation in genes but test cost is relatively expensive for several patients in developing countries like India. Genetic test takes between 2 and 4 weeks to decide the cancer. The time duration suffers the prognosis of genes because some patients have high rate of cancerous cell growth. In the research work, a cost and time efficient method is proposed to predict the gene expression level on the basis of clinical outcomes of the patient by using machine learning techniques. An improved SVM-RFE_MI gene selection technique is proposed to find the most significant genes related to breast cancer afterward explained variance statistical analysis is applied to extract the genes contain high variance. Least Absolute Shrinkage Selector Operator (LASSO) and Ridge regression techniques are used to predict the gene expression level. The proposed method predicts the expression of significant genes with reduced Root Mean Square Error and acceptable adjusted R-square value. As per the study, analysis of these selected genes is beneficial to diagnose the breast cancer at prior stage in reduced cost and time.

## 1 Introduction

**Figure 1:**
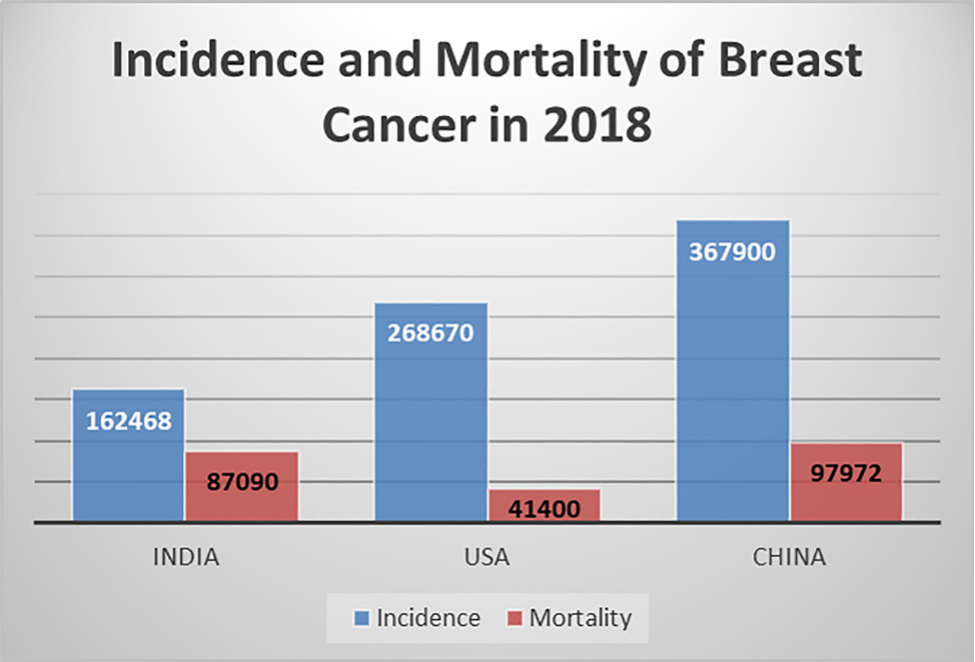
Breast cancer statistics [3], [4], [5], [6].

Breast cancer is a genetic disease in which cells in the breast multiply uncontrollably and become abnormal to generate a tumor. It develops as a result of genetic damage or change (mutation) in cells functioning [1]. As per the study [2], USA, China and India collectively account almost one third of global breast cancer cases whereas India has high mortality rate and low incidence rate in comparison to China and USA [3], [4], [5], [6] as shown in Figure 1. In 2017, India had the highest mortality rate globally in breast cancer. In 2019, 268,600 new cases are estimated of invasive breast cancer among women and around 41,760 women died from breast cancer in 2019. The major reasons of increased mortality in India are the diagnosis of cancer at last stage, inadequate screening, high-cost of screening and lack of required prevention facilities [7]. According to Ferlay et al. [8], In India, one woman is detected with breast cancer, in every 4 min and one woman dies because of breast cancer, in every 8 min. Therefore, advance methods are essential to diagnose the breast cancer at early stage in India.

**Figure 2:**
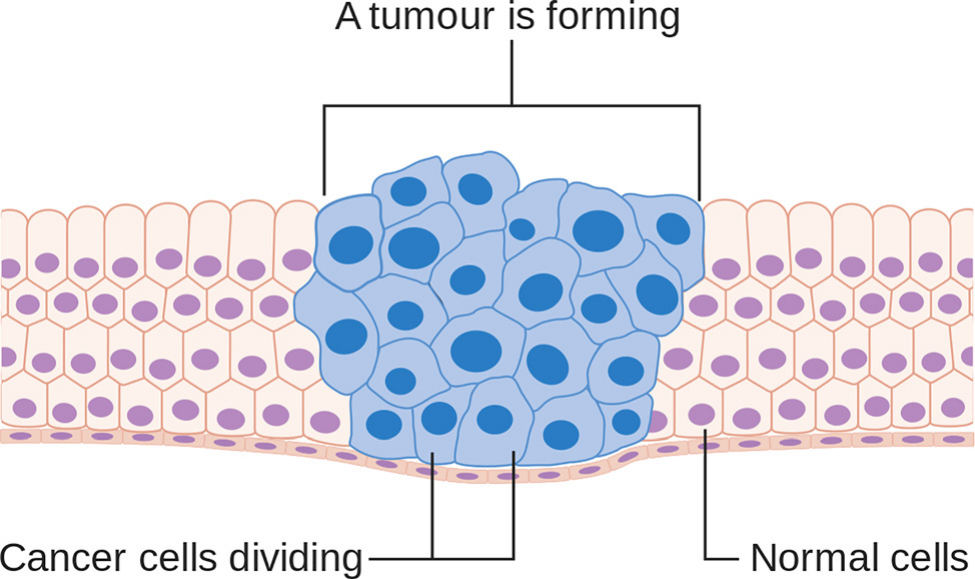
Cancer cells keep on forming the tumor [Cancer Research UK, CC BY-SA 4.0].

Genetic test is an important tool to diagnose the cancer. It is basically a DNA sequencing test that compares the sequence of DNA in normal cells with cancerous cells [9]. A genetic test predicts the prognosis of genes precisely but it is expensive and time taking process to reach the final result in developing countries like India [10]. According to Rajiv Sarin [11], The cost of each reliable genetic test in India is expensive for several families. At present, no government hospital is providing genetic test for cancer, patient has to bear all the expense. Commercial labs give the test report between 2 and 4 weeks but research centers take minimum 4 weeks or more for final report [10]. Normal cells function properly and repair themselves but cancer cells are dented cells, they do not repair themselves and assembled with the boundary of tumor [12] consequently the duration of final gene report can affect the neighbor tissues as shown in Figure 2. So, a novel test is required that can provide genetic test report in reduced cost and time as time is an imperative factor in making decisions for breast cancer.

In current clinical perspective, biomarkers are involved to diagnose the cancer at the cellular level [13], [14], [15]. In clinical test, tissues of the suspected body parts are examined to provide evidence of the disease. It is based on certain biomarkers such as blood presser, dimension of tumor, Progesterone Receptor and Estrogen Receptor status of tumor [16]. Recent research has revealed that these biomarkers help in the prognosis and diagnosis of cancers [17]. The important part of clinical test is the understanding of alteration occurring in the cancer cells at cellular level.

In current state, these genomic and clinical test reports are available in the form of datasets The clinical biomarkers have been generated from gene expression data, for intense 70-gene [18], and 76-gene [19] signatures, and clinical data for example Nottingham Prognostic Index (NPI) [20] and Adjuvant Online! tools [21]. Many researchers have aimed at training model by combining both the datatypes such as clinical data and gene expression. From the past decade, researchers are applying Machine Learning to diagnose the cancer [22]. Machine learning is a field of Computer Science. It takes decision on the basis of past experiences using statistical and computational techniques [23].

In the proposed model, Machine Learning based SVM-RFE_MI technique is proposed for gene selection. It is an improved hybrid model of SVM-RFE and Mutual Information techniques that provides the significant genes associated with breast cancer. Along with this, clinical evidence of breast cancer patient is used to predict the expression level of selected genes by applying Least Absolute Shrinkage Selector Operator (LASSO) regression technique. The goal of lasso regression is to attain the subset of predictors that reduces the prediction error for a measurable response variable.

The research work is described in four sections, next section describes the related work, second section deals with the methods and materials used in the work, third section deals with the experimental results and fourth section describes the conclusion of the proposed work followed with reference section.

## 2 Related work

Breast cancer is a genetic disease that is diagnosed by examining the mutations in genes. Gene expression data contains genetic information of patient that is beneficial to prior diagnose the disease. In the past decades, several researchers have been found out the insight of genomic dataset. The study is presented in Table 1.

**Table 1:** Study of correlation of gene expression and clinical dataset.

S. No.	Methodology	Problem	Dataset	Result
1.	Integration of clinical and gene expression data [22].	Predicting breast cancer outcome	70-gene Vijver dataset, 76-gene data and Nottingham Prognostic Index NPI clinical data	90% accuracy
2.	Gene expression profiling predicts clinical outcome of breast cancer [18].	Prediction of clinical report	Van’t Veer microarray data	94% accuracy
3.	Evaluating the clinical validity of gene-disease associations [24].	Clinical report validity using gene data.	LOVD v.2.0: The gene variant databases	87.1% accuracy
4.	Gene expression correlates of clinical prostate cancer behavior [25].	Cancer behavior estimation	Clinical data and oligonucleotide array-based expression data for 8 normal and 27 prostate tumors	90% accuracy
5.	Stromal gene expression predicts clinical outcome in breast cancer [26].	Prediction of clinical report	Gene expression data of 110 genes by SAM analysis	14.54 Hazard ratio
6.	Biological analysis of gene expression and clinical variables [27].	Finding of novel biomarker FZD1 for patients with Kashin-Beck disease	Gene expression data	FZD1 perform better in comparison to others

Machine learning application in cancer detection and gene selection				
S. No.	Machine learning technique	Problem	Dataset	Result
1.	Neighborhood rough sets and entropy measures, KNN, C4.5, SVM [28].	Gene selection for tumor classification	SRBCT gene expression data	SVM performs better with 92% accuracy
3.	Multiple SVD-RFE [29].	A fast gene selection method for multi-cancer classification.	Microarray datasets	90% accuracy
4.	Consensus SVM-RFE [30].	Sparse and stable gene selection	Three benchmark microarray datasets, the Golub dataset, the Alon dataset and the Nutt dataset	The 10-fold CV error is 4% at stabilized f = 0.9
5.	NCMIGS: NCMI-based gene selection algorithm [31].	Gene selection and cancer classification algorithm.	Six microarray datasets used in experiments.	LOOCV classification accuracies of k- NN is 94.04% on EGS-NCMI
7.	SVM [32].	Predict breast cancer susceptibility using nucleotide polymorphisms	RNA microarray data	69% predictive power
6.	Implemented 17 SVM classifier model using LIBLINEAR [33].	Classify the primary sites of cancer	Clinical dataset	62% accuracy
8.	Support vector machine (SVM) [34].	Breast cancer prognosis	Microarray dataset	94.5 % predictive power
9.	Non-linear biomarker Association Network (NLN) compares with five previous classification methods, KNNs, FDA, SVMs, BN, with linear and non-linear kernel methods are performed, which further confirm the performance of the classifier [35].	A neural network-based biomarker association information extraction approach for cancer classification.	Protein expression dataset, Nasopharyngeal carcinoma (NPC), and three gene expression datasets, leukemia, colon and breast	NLN classifier performs better on leukemia and colon dataset where SVM performs better on breast cancer dataset with 100% accuracy
10.	Mutual information [36].	Mutual information relevance networks: Functional genomic clustering using pairwise entropy measurements	RNA expression data	0.2 error rate
11.	Combine the traditional distance criteria and mutual information criterion such as the fuzzy membership metric and the Euclidean distance [37].	Gene clustering	Gene-wide expression data from DNA microarray hybridization	92% accuracy
12.	ANN, DT, random Forest (RF), Naïve Bayes Classifier (NBC), SVM and KNN [38].	Breast cancer prediction	Wisconsin breast cancer data	SVM performs better with 97% accuracy.

As per the study, researchers have utilized the correlation of genomic and clinical information in their research. Gene expression has power to predict the clinical information as well as clinical data also has high correlation with gene expressions.

Researchers have also applied machine learning in the gene selection and cancer detection. SVM is the most widely used technique in the cancer detection due to its large margin and kernel facility to discriminate the mutations of gene. Mutual Information found extensively used ranking method because it measures the general dependency among gene random variables.

## 3 Materials and methods

This section deals with dataset, methods, work flow and performance parameter to evaluate the proposed work. An authentic breast cancer microarray dataset is used in the research.

### 3.1 Dataset

In this experiment Van’t Veer microarray data is used [39]. The dataset is available to download from the research submitted to Nature by Laura J. van’t Veer and other authors. This dataset has sample of 117 patients suffering from primary breast cancer. The dataset contains six related files such as:

ArrayData_less_than_5yr.xls
  Data of 34 patients having less than 5 years disease-free survival.
ArrayData_greater_than_5yr.xls
  Data of 44 patients having greater than 5 years disease-free survival.
ArrayData_19samples_.xls
  Data of 19 additional patients profiled.
ArrayData_BRCA1.xls
  Data of 18 BRCA2 and 2 BRCA1 patients.
ArrayNomenclature_methods.doc
  Report of the derivation of illustrative GenBank accession numbers provided for the EST contig assemblies on the array.
ArrayNomenclature_contig_accession.xls
  Identifiers for EST contig assemblies used in the array design and representative GenBank accession numbers for each.

Every datafile have the listed columns:

*Systematic name*: The name given to each sequence or gene.
*Gene name*: common name given by scientist.
*Gene description*: Report of each gene’s function.

Each tumor sample have information in three columns. Two microarray barcodes are given for each tumor sample profiled, with a description of the sample such as disease-free survival, patient age, sample number etc. Each gene profile contains three fundamental values such as Log10(ratio), Log10(Intensity) and *p*-values.

In the research work, all the related files are applied using intensity values of genes for analysis. It is basically, the geometrical mean intensity for both red (cancerous cells) and green (normal cells) channels for a given probe on the microarray chip. As per study, quality data are resulting from the genes related to the greatest signal intensity. Therefore, genes accompanying with truncated mean intensity values may not be allocate low *p*-values, though the mean ratio is far from 1.

It has two classes, good and bad prognosis of genes. Clinical dataset contains 11 attributes and genomic dataset contains 24,500 genes. For the analysis, Dataset is divided in two parts, 70% data used for training part and 30% data is used for testing part.

### 3.2 Proposed method

In the research work, genomic and clinical both datasets are processed to impute missing values using mean technique. Simple techniques such as imputing the missing values by median or the mean values performed similar to more complex approaches [40]. The attributes containing 80% missing values are removed from dataset to make the model unbiased. Remaining missing values are reconstructed. Afterward, features selection techniques are applied on genomic data to get the most relevant genes that contain more predictable power and higher variance in the dataset.

The accuracy of cancer detection mainly depends on the biological significance of genes [41]. So, gene selection is the vital stage for cancer detection on the basis of gene expression data. In microarray data, few genes contain highly correlated expression level, which shows an imperative role in biological evolution. When these correlated genes are sited on the biological trail, this correlation is more definite [42]. Therefore, traditional feature selection techniques overlook the association between genes, and select only few among these high correlated genes. The inappropriate genes not only add extra difficulties to find the informative genes, but lower the detection performance also [43].

In the research work, SVM-RFE_MI technique is applied that is the improved hybrid model of SVM-RFE and mutual information technique for gene selection.

#### 3.2.1 SVM-RFE_MI gene selection technique

A Support Vector Machine (SVM) is the machine learning technique which performs classification by finding the optimum hyperplane that maximizes the distance margin between the two classes [34]. The extreme points in the data sets that state the hyperplane are the support vectors. The hyperplane is the set of points $\bar{x}$ satisfying

(1) $$\bar{w}\cdot \bar{x}-b=\left(0\right)$$

where $\bar{w}$ represents the normal vector to the hyperplane identical to Hesse normal form, excepting that $\bar{w}$ is not certainly a unit vector. The parameter $\frac{b}{\left|\left|\bar{w}\right|\right|}$ governs the balance of the hyperplane from the origin towards the normal vector. SVM extend with the hinge loss function that works well when datapoints are not linearly separable. Hinge loss function:

(2) $$\max\left(0,1-y_{i}\left(\bar{w}\cdot \bar{x}-b\right)\right)$$

where *y*_*i*_ is the *i*th target *y*_*i*_ and $\left(\bar{w}\cdot \bar{x}-b\right)$ is the present output. Hinge loss function becomes zero if $\bar{x}$ lies on the right side of the margin. the function’s value is proportionate to the distance from the margin If it lies on the incorrect side of the margin. Then the Hinge loss is need to minimize:

Then it wishes to minimize

(3) $$\left[\frac{1}{n}\sum_{i=1}^{n}\max\left(0,1-y_{i}\left(\bar{w}\cdot \bar{x}-b\right)\right)\right]+\lambda {\left|\left|\bar{w}\right|\right|}^{2}$$

where *λ* limits the trade-off between growing the margin size and confirming that the $\bar{x}$ lie on the right side of the margin. Therefore, appropriately small values of *λ* will become negligible. That help to regularize the non-linearity of dataset.

It has the kernel facility that helps to capture much more complex relationships between your datapoints and perform difficult transformations.

SVM-RFE (Recursive Feature Elimination) is an SVM based wrapper feature selection method. It selects the features by the help of classification method [44]. RFE needs training of multiple classifiers on subgroups of features of reducing size. The training time grows linearly with the number of classifiers to be trained. Analysis of one part can iterates for next parts so that complete matrix does not need to be re-computed completely. In each iteration, one feature eliminates that contain low weight afterward the partial scalar products of the rejected features can be eliminate and the coefficients reset to their previous value [45] but it assigns the equal weight to correlated attributes [46]. So, the gene selection process treats all the correlated gene in a same manner. To overcome this issue Mutual Information (MI) ranking technique is combined with SVM-RFE.

Mutual information is a measure between two arbitrary variables A and B, that computes the amount of information obtained in one random variable, using the other random variable. In genomics, the mutual information is given by:

(4) $$I\left(A;B\right)=\int_{A}\int_{B}p\left(a,b\right)\log\frac{p\left(a,b\right)}{p\left(a\right)p\left(b\right)}dady$$

where *p*(*a*, *b*) represents the joint probability density function of A and B, and where *p*(*a*) and *p*(*b*) are the marginal density functions. MI technique governs how similar the joint distribution *p*(*a*, *b*) is to the products of the factored marginal distributions. If A and B are completely independent, then *p*(*a*, *b*) would equal *p*(*a*)*p*(*y*), and this integral would be zero. So, MI [47] provides the total interaction information between random variables, which is beneficial to find out the interaction between two correlated genes.

In case of feature selection, mutual information maximizes between the subset of selected features AS and the target variable *b*.

(5) $$\tilde{S}=\arg\max I\left(\text{AS};b\right)$$

SVM-RFE_MI feature selection process follows four steps:

(1) Pre-processing of datasets to be categorize,
(2) analysis of weight of each feature, and
(3) the deletion of the feature of minimum weight
(4) Rank evaluation as shown below [48]:
  (a) Input
    Training Example: *X* = ${\left[x^{1},x^{2},x^{3}\ldots \ldots x^{n}\right]}^{T}$

    Class Labels: *Y* = ${\left[y^{1},y^{2},y^{3}\ldots \ldots y^{n}\right]}^{T}$

    The current surviving feature set: *c* = [1, 2, 3 … *n*]
    Reduced Feature ranked list: *L* = [ ]
  (b) Feature Sorting
    Repeat the process till *L* = [ ] is received
    A new training data matrix as per the remaining features: *X*1 = *X*(:*c*)
    Classifier used: *α* = SVM-train (*X*, *Y*).
    Calculation of weight: *w* = ∑ (*k* ^*x*^ *k* ^*y*^ *k* ^*x*^) *k*
    Calculate the features of the minimum weight: *m* = arg min (*c*)
    Updating the sorted feature list: *L* = [*c*(*m*), *L*].
    Eliminating the features with minimum weight: *c* = *c* (*L*: −*L*, *m* + *L*: length(*c*))
  (c) Reduced Set: This step contains sorted feature list. In each iteration, the feature containing minimum (*w*_i_)^2^ weight is eliminated on the basis of prediction accuracy. SVM-RFE is repeated until a feature sorted list F is achieved. The sorted list F contains 100 relevant features among 24,500 but the feature list contains the correlated attributes having high weights because SVM-RFE assign the equal weights to related attributes. So, an MI ranking method is applied to find the significant genes.
  (d) Rank evaluation of genes: Revised MI technique is applied to find the rank of each gene and provide the most explainable genes. For MI, first entropy is calculated for each gene (*A*) in F with mass probability *p*(*A*(*i*)) = Pr{*A* = *A*(*i*)}, *A*(*i*)⋲*A* such as:

(6) $$H\left(A\right)=-\sum_{i=1}^{n}p\left(A\left(i\right)\right)\mathrm{lo}g_{2}\left(p\left(A\left(i\right)\right)\right)$$

Now, the joint probability is calculated to find the information between two genes *A* and *B*, with joint mass probability *p*(*A*(*i*), *B*(*j*)). Joint mass probability is the sum of uncertainty contained by two genes; it is defined as:

(7) $$H\left(A,B\right)=-\sum_{i=1}^{n}\sum_{j=1}^{n}p\left(A\left(i\right),B\left(j\right)\right)\cdot \mathrm{lo}g_{2}\left(p\left(A\left(i\right),B\left(j\right)\right)\right)$$

In the proposed technique, conditional probability is employed to find the remaining uncertainty of pair of genes that helps to find all the by using equation (8). It estimates the probability of getting different pattern in each gene that provides the whole-distribution view of data by interconnecting all the variables:

(8) $$H\left(A|B\right)=\sum_{j=1}^{n}p\left(y\left(i\right)\right)\cdot H\left(A|B=B\left(j\right)\right)$$

Where, 0 < *H*(*A*│*B*) < *H*(*A*)

MI is useful for feature selection because it provides the relevance of a feature subset with respect of output. MI is linearly related to entropy and defined as follows:

(9) $$I\left(A;B\right)=\left\{ \begin{array}{c} H\left(A\right)-H\left(A|B\right)\\ H\left(B\right)-H\left(B|A\right)\\ H\left(A\right)+H\left(B\right)-H\left(A,B\right) \end{array}\right.$$

So, mutual information between two gene is calculated using equation (2)–(4) as follows:

(10) $$I\left(A;B\right)=\sum_{i=1}^{n}\sum_{j=1}^{n}p\left(A\left(i\right),B\left(j\right)\right).\log\left(\frac{p\left(A\left(i\right),B\left(j\right)\right)}{p\left(A\left(i\right)\right).p\left(B\left(j\right)\right)}\right)$$

*I*(*A*; *B*) becomes zero when *A* and *B* are statistically independent. It provides the rank of each attribute between 0 and 1. The improved technique provides the gene that has scored rank between 0.9 and 1. These are the expressive genes among all. In this way, 20 most relevant genes are selected among 100 genes and feature list *F* is updated by storing these gene as shown in Table 2.

**Table 2:** Rank wise list of top 20 selected genes.

Accession no.	Rank	Rank	
NM_003158	AURKA	1	
NM_000599	IGFBP5	0.98	
NM_000849	GSTM1	0.976	
NM_000017	ACADS	0.972	
NM_000507	FBP1	0.962	
Contig37598	MMSDH	0.956	
NM_003234	TFRC	0.952	
NM_002358	MAD2L1	0.947	
NM_004358	CDC25B	0.945	
NM_014754	PTDSS1	0.941	
Contig41413_RC	RRM2	0.938	
NM_001333	CTSL2	0.936	
NM_014363	SACS	0.930	
NM_001905	CTPS	0.929	
D25328	PFKP	0.926	
NM_004052	BCL2	0.925	
NM_000158	GBE1	0.921	
NM_003376	VEGF	0.917	
NM_003748	ALDH4	0.912	
AF148505	MMSDH	0.905	

#### 3.2.2 Explained variance

After feature selection, explained variance of each gene is calculated using Principal Component Analysis (PCA) [49]. PCA incorporate with the total variation in the dataset and transform the original attributes in the reduced set of linear combination [50]. The reduced set still contains foremost information of dataset. PCA is generally applied when the concern is to find the minimum number of features that has the higher number of variances. PCA extracts the features in the form of Principal Component (PC), where PC is the linear combination of all genes stored in *F*.

Certainly, the coefficients č1, č2, č3 ……… č*n* in the first principal component *PC*_1_:

$PC_{1}=\text{č}1G_{1}+\text{č}2G_{2}+\text{č}3G_{3}+\ldots +\text{č}nG_{n}$ gives you the maximum value (*S*) such as:

(11) $$S=\sum_{i=1}^{n}V_{i}^{2}\left(G_{i}|PC_{1}\right)$$

where *V*_*i*_ is the eigenvector calculated from the covariance matrix of *F* for *G*_*i*_. In covariance matrix, element at (*i*, *j*) entry is the covariance between the genes (*G*_*i*_, *G*_*j*_) of *F*. It is calculated as:

(12) $$K_{GG}=COV\left[G_{i},G_{j}\right]=E\left[\left(G_{i}-E\left[G_{i}\right]\right)\left(G_{j}-E\left[G_{j}\right]\right)\right]$$

where E denotes the mean of the argument. In eq. (11), maximum S is taken over all possible linear combinations. In the research work, top six genes are selected with 75% explained variance because variance was slightly increasing after six genes that will lead to increase the computation as shown in Table 3. As per the study, acceptable cumulative variance is 70% [51], [52] that shows the explained ratio of selected genes is acceptable.

**Table 3:** Top 6 genes have 75% explained variance ratio.

Gene	Explained variance	Cumulative variance	Description
AURKA	44	44	Serine-threonine kinases
GSTM1	10	55	Glutathione S-transferase mu 1
IGFBP5	8	62	Growth factor binding protein 5
BCL2	5	67	Apoptosis regulator
VEGF	4	71	Vascular endothelial growth factor
RRM2	4	**75**	Ribonucleotide reductase
			Regulatory subunit M2

Total cumulative variance is 75 of all the selected relevent genes. Bold number is representing the contribution of each gene in the total variance.

#### 3.2.3 Prediction of gene expression level

Microarray expression analysis is used to determine global biological differences lie beneath common pathological features of *x* cancer and to categorize genes that predicts the clinical behavior of the disease.

As per the study, the clinical data contains biomarkers that has supremacy to classify patients in subcategories, and then train a gene expression predictor model in each of the category. Both the datasets are correlated with each other. From past decades, researchers are training the model using both the datasets.

In the research work, clinical outcomes are utilizing to predict the gene expression level of the patient. Here, regression technique is applied to predict the expression level. Regression technique provide the outcome in the same format of the gene expression (continuous). It predicts the value on the basis of relationship between independent variable and target variable.

In the work, most widely used regression techniques Lasso, Ridge are applied to find the accurate prediction of gene expression level.

##### 3.2.3.1 Lasso regression

LASSO provides higher prediction accuracy and increase model interpretability. It is similar to linear regression with the advantage of shrinkage [53]. In shrinkage, data points are shrunk towards the absolute mean. Lasso regression provides a reduced set of features. This technique is suitable for the dataset containing multicollinearity. It performs *L*1 regularization and adds penalty to the loss function. This penalty contains the absolute value of the coefficients as shown in equation (8).

(13) $$\theta^{\text{lasso}}=\text{min}\sum_{i=1}^{n}{\left(y_{i}-\bar{\text{y}}\right)}^{2}+\lambda \sum_{j=0}^{k}\left|\theta_{j}\right|$$

Here the *λ* is a turning factor that controls the strength of penalty. In this research work, mean square error induced from 10-fold cross validation is used to estimate the expected generalization error for turning factor *λ*. So, *λ* is wisely chosen to minimize this estimation which is 0.0032. Standard results of *λ* are as follows:

When *λ* = 0: Same coefficients are selected as simple linear regression,
When *λ* = ∞: No attribute is selected, all coefficients are zero,
When 0 < *λ* < ∞: We get reduced coefficients between 0 and *n* where *n* is linear regression coefficients.
when *λ* will increase, bias of the model will increase.
when *λ* will decrease, variance of the model will increase.

##### 3.2.3.2 Ridge regression

Ridge regression basically is an instance of linear regression with regularization. In a multiple linear regression, there are various variables to process [54]. Sometimes, it creates a problem of selecting the wrong variables for the machine learning, which provides the unwanted output as a result. Ridge regression is used to overcome this problem. Ridge regression is a regularization technique, that add an extra tuning parameter and optimized to balance the outcome of multiple variables in linear regression.

The cost function for ridge regression:

(14) $$\theta^{\text{Ridge}}=\text{min}\left(\|Y-{X\left(\theta \right)\|}_{2}^{2}+ƛ{\|\theta \|}_{2}^{2}\right)$$

The tuning parameter ƛ is involved in the ridge regression model as part of regularisation. Decreasing the *ƛ*, the solutions adapt to least square method. Increasing the value of *ƛ*, the residual sum of squares tends to be zero. Unlike Las it does not delete the high collinear parameters it just shrinks the parameters.

Gene expression of the top 6 significant genes are predicted individually using LASSO and Ridge regression technique [53].

**Figure 3:**
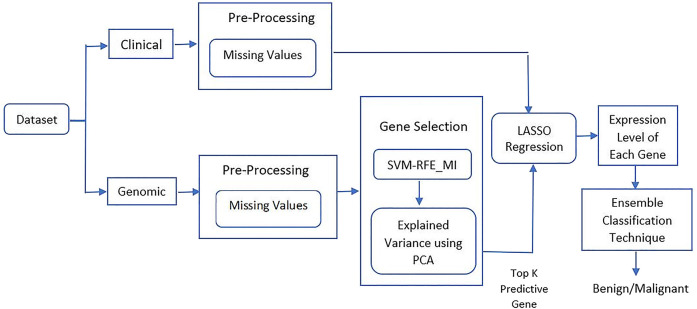
Work flow of the proposed work.

To process the regression technique, genomic dataset is randomly divided in training and testing. Training data contains 70% samples to train the regression model and test data contains 30% samples to test the model. Individual genes are selected as dependent variable and all clinical attributes are selected as an independent variable. So, a multivariate analysis is performed to predict the expression level of significant genes. Figure 3 shows the overall workflow of the research work.

In the work, experiment is performed on Apache Spark data processing engine and Python programming language.

### 3.3 Apache spark

It is an open source data processing engine. It is a parallel framework to process a massive amount of data. It is a general-purpose cluster computer system [55].

Apache Spark can handle both real time and batch analytics. It can process the data kept in several file systems like Hadoop Distributed File System (HDFS), Relational and NoSQL databases. It performs in-memory processing of data to improve the performance of analytics.

### 3.4 Performance parameters

In the research, proposed work is compared in two aspects one on the basis of error and other to examine the prediction closeness to the predictor. Therefore, two performance metrics are used to validate the regression model such as: Root Mean Square Error (RMSE) and adjusted R-squared [56]. Adjusted R-squared is applied for multivariate linear regression to find out the variance explained by the model and RMSE is applied to find the standard deviation of the prediction errors.

#### 3.4.1 Adjusted R-squared

It is a statistical metrics and calculated as the ratio of variance of a dependent variable explained by the independent variable in regression model [57]. The adjusted R-squared $\left({\bar{R}}^{2}\right)$ considers the predictors that have the significance and directly affect the dependent variables [58]. Therefore, it works well in the multivariate regression models. It is calculated as:

(15) $${\bar{R}}^{2}=1-\left(1-R^{2}\right)\left[\frac{N-1}{N-\left(I+1\right)}\right]$$

where, *N* denotes the total number of samples and I represents the total independent variable. The rage of adjusted R-Squared value is between 0 and 1 like $0\leq {\bar{R}}^{2}\leq 1.$ If ${\bar{R}}^{2}$ is close to 1, it means predicted regression line is equal to the original regression line.

#### 3.4.2 Root mean square error

In the regression model, regression line predicts the average of dependent variable *y* on the basis of *x* values [59]. RMSE is used to find the correctness of *y* values using the average. It is calculated as:

(16) $$\text{RMSE}=\sqrt{\frac{1}{n}}\sum_{j=1}^{n}{\left(Y_{i}-{\hat{Y}}_{i}\right)}^{2}$$

where, *n* represents the total number of samples, *Y*_*i*_ is observed value and ${\hat{Y}}_{i}$ is predicted value. Lower value of RMSE represents the improved performance of the model.

## 4 Results and discussion

In this section, the outcomes of performed experiments are discussed. In the experiment, SVM-RFE_MI technique is proposed to find the significant genes of breast cancer. This technique applied on genomic data containing 24,500 genes and provides the output of 20 significant genes as presented in Table 2.

These selected genes are relevant breast cancer genes. Afterward, higher explained variance genes are required to make the model efficient. Genes containing high explained variance are more predictive. So, PCA is applied to find the explained variance in selected genes. It provides top 6 genes with total 75% variance as shown in Table 3. The description of genes are as follows.

Gene 1- AUREKA: It is a kinase, that is important for cell division. Its main function is regulating the mitosis specifically chromosomal segregation. The mutation in AURKA kinases leads to failure of cell division and harm the progression of cells [60], [61].
Gene 2- GSTM1: It is antioxidant, it converts free radicals into molecules. Mutations of this gene leads to unstable the free radicals. These free radicals damage the cells and converts into cancerous cells [62].
Gene 3- IGFBP5: It is an Insulin-like protein-binding growth factor that plays a vital role in cell growth, differentiation and apoptosis. Its key role is cell regulation and breast tissue development. IGFBBPS mutation could lead to differentiation of breast tissue and development of cancer [63].
Gene 4- BCL2: It is Apoptosis Regulator. Cancer cell depends on this gene to survive. BCL2 needs to remove so that cells can undergo programmed cell death [64].
Gene 5- VEGF: It is an endothelial vascular growth factor. It induces mitogenesis, survival of endothelial cells, stromal degradation and vascular permeability. Overexpression of VEGF lead to tumor development and neovascularization [65].
Gene 6- RRM2: It is ribonucleotide reductase regulatory subunit, which catalyzes the development of ribonucleotide deoxyribonucleotides. RRM2 regulate the cell cycle by synthesis and degradation of DNA and RNA. Inhibition of this enzyme in cancer patients considerably reduce cell cycle gene expression [66].

These genes are verified from Cancer Genetics Web [67]. Researchers are targeting these selected genes for breast cancer detection. These selected genes contain higher variance and predictiveness to predict the breast cancer at early stage. So, if experts target these genes, the cancer will be diagnosed at prior stage.

These genes are not correlated with each other as sown in Table 4. So, the values of each gene predicted individually. In the research work, a multivariate LASSO regression model is applied to predict the gene expression. So, the attributes of clinical data considered as independent variable and each gene is considered as a dependent variable. The prediction results on test data are represented in Table 5 and Table 6. Table 5 represented results on the basis of ${\bar{R}}^{2}$ and RMSE evaluation parameters using LASSO technique and Table 6 represented results on the basis of ${\bar{R}}^{2}$ and RMSE evaluation parameters using Ridge technique.

**Table 4:** Correlation matrix of top 6 genes.

Gene	AURKA	GSTM1	IGFBP5	BCL2	VEGF	RRM2
AURKA	1	0.3	−0.01	−0.22	−0.14	−0.4
GSTM1		1	−0.25	−0.43	−0.48	−0.3
IGFBP5			1	0.29	0.29	0.19
BCL2				1	0.5	0.42
VEGF					1	0.44
RRM2						1

**Table 5:** Evaluation of predicted genes using LASSO on the basis of ${\bar{R}}^{2}$ and RMSE.

Gene	${\bar{R}}^{2}$	RMSE
AURKA	0.9	0.13
GSTM1	0.78	0.35
IGFBP5	0.82	0.28
BCL2	0.89	0.23
VEGF	0.91	0.19
RRM2	0.85	0.20

**Table 6:** Evaluation of predicted genes using Ridge on the basis of ${\bar{R}}^{2}$ and RMSE.

Gene	${\bar{R}}^{2}$	RMSE
AURKA	0.85	0.15
GSTM1	0.73	0.34
IGFBP5	0.76	0.30
BCL2	0.79	0.26
VEGF	0.88	0.23
RRM2	0.82	0.24

**Figure 4:**
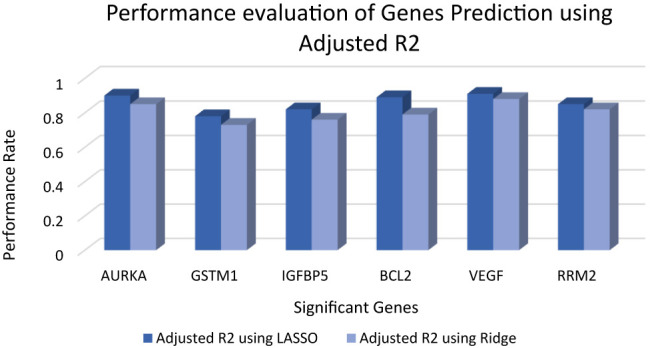
Performance evaluation of gene prediction using LASSO regression and ridge regression on the basis of ${\bar{R}}^{2}$ .

**Figure 5:**
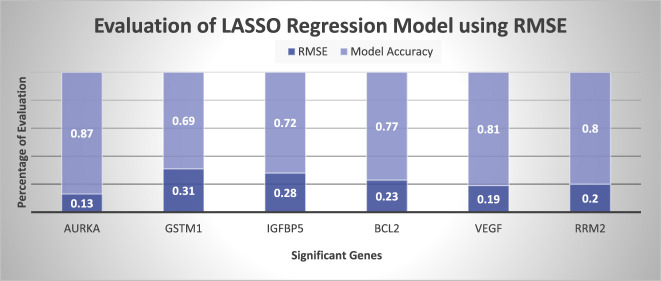
Performance evaluation of LASSO regression model using RMSE.

**Figure 6:**
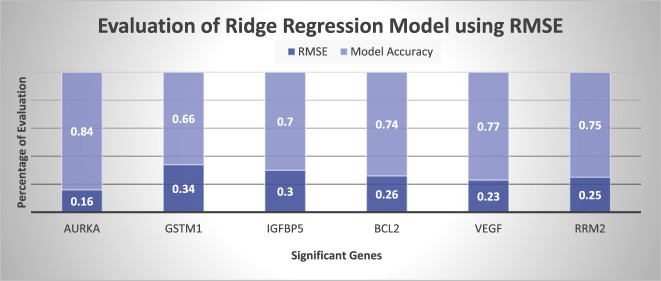
Performance evaluation of ridge regression model using RMSE.

The graphical representation of prediction accuracy of Lasso and Ridge regression model are represented in Figure 4 in terms of ${\bar{R}}^{2}.$ Figure shows that LASSO regression model is more accurate then Ridge regression model because LASSO contains only independent attributes whereas Ridge contains all the attributes contains low weight of correlative attributes. It represents that LASSO is preferred over Ridge regression model. Figure 4 represents the prediction of LASSO regression model on the basis of ${\bar{R}}^{2}$ is minimum 7.8 for GSTM1, as per the Study [68] ${\bar{R}}^{2}$ should be greater than 0.7 and close to one. That shows the model has acceptable ${\bar{R}}^{2}$ error. Evaluation of predicted gene expression using LASSO in terms of RMSE is represented in Figure 5. It shows that regression model has minimum 0.13 RMSE in AURKA gene. Whereas GSTM1 has 0.35 RMSE which is maximum error. According to the study, RMSE should be less than 0.5 or close to zero for a good model [69]. So, the proposed experiment shows the acceptable RMSE error to predict gene expression. Figure 6 represents the evaluation of Ridge regression model in terms of RMSE. It represents that error of ridge model is larger than LASSO regression model. This method will generate gene expression which is beneficial to every breast cancer patient.

## 5 Conclusion

Breast cancer is frequent occurring cancer among women. It occurs due to the mutation in genes, therefore genetic test is preferred to identify the gene mutation. Genetic test report is accurate to detect the tumor stage but the test is expensive and time consuming in developing countries like India. In this research work, a novel test method is generated to predict the gene expression in reduced cost and time. This test method generates the expression of most significant genes from clinical outcome and provides the prognosis stage of cancer. This test minimizes the risk of breast cancer by identifying the mutations of gene at early stage.

The method is generated by the help of proposed SVM-RFE_MI gene selection technique and LASSO regression technique. It is evaluated by using Root Mean Square Error and adjusted R-Squared performance parameters. The results show that adjusted RMSE and R-Squared values lies within standard acceptable range. It represents that the test method has good prediction accuracy.

This test method will provide outcome immediately after final clinical report with no cost. It is useful for all the patients suffering from breast cancer. The proposed test method leads to reduce the mortality by identifying the cancer at earliest phase.
